# Zika epidemic and microcephaly in Brazil: Challenges for access to health care and promotion in three epidemic areas

**DOI:** 10.1371/journal.pone.0235010

**Published:** 2020-07-07

**Authors:** Paulo Cesar Peiter, Rafael dos Santos Pereira, Martha Cristina Nunes Moreira, Marcos Nascimento, Maria de Fatima Lobato Tavares, Vivian da Cruz Franco, José Joaquin Carvajal Cortês, Daniel de Souza Campos, Christovam Barcellos

**Affiliations:** 1 Laboratory of Parasitic Diseases, Institute Oswaldo Cruz/Fiocruz, Rio de Janeiro, Rio de Janeiro, Brazil; 2 National Institute of Women, Children and Adolescent Health Fernandes Figueira/Fiocruz, Rio de Janeiro, Rio de Janeiro, Brazil; 3 Department of Health Administration, Planning and Management, Sergio Arouca National School of Public Health/Fiocruz, Rio de Janeiro, Rio de Janeiro, Brazil; 4 Institute Leônidas & Maria Deane/Fiocruz Amazonia, Manaus, Amazonas, Brazil; 5 Health Information and Communication Institute/Fiocruz, Rio de Janeiro, Rio de Janeiro, Brazil; VA-MD College of Veterinary Medicine, UNITED STATES

## Abstract

Since 2015 Brazil has experienced the social repercussions of the Zika virus epidemic, thus raising a debate about: difficulties of diagnosis; healthcare access for children with Zika Congenital Syndrome (ZCS); the search for benefits by affected families; social and gender inequalities; and a discussion on reproductive rights, among others. The objective of this article is to analyse access to specialized health services for the care of children born with ZCS in three North-eastern states of Brazil. This is an exploratory cross-sectional study which analyses recorded cases of microcephaly at the municipal level between 2015 and 2017. Most of the cases of ZCS were concentrated on the Northeast coast. Rio Grande do Norte and Paraiba had the highest incidence of microcephaly in the study period. The states of Bahia, Paraiba and Rio Grande do Norte were selected for their high incidence of microcephaly due to the Zika Virus. Socio-territorial vulnerability was stratified using access to microcephaly diagnosis and treatment indicators. The specialized care network was mapped according to State Health Secretaries Protocols. A threshold radius of 100 km was stablished as the maximum distance from municipalities centroids to specialised health care for children with microcephaly. Prenatal coverage was satisfactory in most of the study area, although availability of ultrasound equipment was uneven within states and health regions. Western Bahia had the lowest coverage of ultrasound equipment and lacked health rehabilitation services. ZCS's specialized health services were spread out over large areas, some of which were outside the affected patients' home municipalities, so displacements were expensive and very time consuming, representing an extra burden for the affected families. This study is the first to address accessibility of children with microcephaly to specialised health care services and points to the urgent need to expand coverage of these services in Brazil, especially in the northeastern states, which are most affected by the epidemic.

## Introduction

Since 2015, the Brazilian population has been living with the social repercussions of the Zika virus (ZIKV) epidemic, which have raised a debate about: diagnostic difficulties; access to care for children with congenital ZIKV syndrome (ZCS); the search for financial compensation by the affected families; social and gender inequalities; a discussion on reproductive rights, among others [1].

Once considered a 'benign' disease—with no major clinical consequences, except for some symptoms such as rashes and oedema–the Zika virus infection became a public health problem after its relationship with microcephaly cases [2,3,4], Guillain-Barré Syndrome and encephalitis had been confirmed [5].

The first suspected cases of Zika were reported in October 2014 in the Brazilian State of Rio Grande do Norte as an outbreak of a rash of unknown origin [6]. In 2015 and 2016 Zika had spread throughout most of the country, with outbreaks in almost every major city of the Northeast. In these regions certain states gained prominence, and the cities with greater resources, but not organized in a network well adapted for the additional demand, became places of confluence of families seeking specialized healthcare and rehabilitation for children born with microcephaly and other diseases related to Zika.

Between January and November 2016, World Health Organisation (WHO) declared a situation of Public Health Emergency of International Concern (PHEIC) due to the rapid spread of the disease and its health and social repercussions, especially the cases of microcephaly.

The Brazilian Primary Health Care (BPH) network had been expanding the coverage of access to health services throughout the country until 2017 [1], mainly through the Basic Health Units (BHU) and the Family Health Strategy (FHS), which together constitute the main gateways to the Unified Health System (UHS). The actions of multidisciplinary family health teams facilitated access to the network and enabled the implementation of health surveillance measures and actions, such as early detection of suspected cases of diseases such as dengue, chikungunya and Zika, the main urban arboviruses in the country, in addition to carrying out actions on maternal and child health in the assigned territories.

WHO recommends that every woman should have at least seven prenatal visits during pregnancy, but since the causal link between microcephaly and ZIKV infection has been established, prenatal consultation procedures must be adapted to changing needs, thus promoting early diagnosis of ZCS and follow-up of pregnant women at risk. Prenatal care practices should now include imaging (ultrasound, computed tomography, and magnetic resonance imaging), laboratory tests for serological diagnosis of ZIKV infections and other diseases that may cause microcephaly (syphilis, toxoplasmosis, among others), complementary laboratory tests, epidemiological investigation of suspected cases, as well as guidance for mothers and relatives [7].

In Brazil, since October 2015, microcephaly attributed to ZIKV infections has been reported in obstetric ultrasound examinations, so prenatal care is essential to prevent complications from exanthematic diseases for mother and baby [8]. Ensuring access to care by a gynaecologist and/or obstetrician trained to identify ZCS-related conditions is paramount for timely diagnosis of the disease and follow-up in suspected cases during pregnancy.

The use of diagnostic support equipment, such as ultrasound, is fundamental for the correct diagnosis of foetal changes, such as those caused by the ZIKV in pregnant women infected before or during pregnancy. WHO recommends three examinations during pregnancy, one in the first trimester (between 11 and 14 weeks), another in the second semester (between the 20th and 24th week), and in the last trimester (between the 32nd and 36th week). However, each pregnancy has its own peculiarities, and it is up to the gynaecologist-obstetrician to determine if there is a need for further examinations. There is scientific evidence that ultrasound examination before the 24th week of pregnancy increases the chances of detecting foetal malformations [9].

Given this situation, two main challenges are posed for the health system: 1—the need to identify, receive, monitor and offer comprehensive treatment to children born during the Zika epidemics and who have neurodevelopmental disorders, whether or not accompanied by other neurological malformation in addition to microcephaly; 2—maintain vigilance over the entire health care and surveillance network to detect early infections of pregnant women by ZIKV and possible malformations and changes in neurodevelopment in new-borns in the coming years, to help forecast the possible return the virus’ circulation in the country.

This article aims to analyse access to specialized services necessary for the integral care of children born with ZCS in the states of Rio Grande do Norte, Paraiba and Bahia in the Northeast region of Brazil most affected by the epidemic.

## Materials and methods

This is an exploratory descriptive study using secondary data. It starts with a health geography approach to analyse access to health resources by children affected by ZCS in three States that presented a high number of cases of microcephaly in the Zika epidemic from 2015 to 2017.

Firstly, to know the spatial distribution of microcephaly by Zika in Brazil, we demanded official access to the Brazilian Ministry of Health to data from Public Health Event Records (PHER). This data was collected and classified in confirmed cases of microcephaly by ZIKV in the years of 2015, 2016 and 2017 and then mapped by municipalities. To maintain personal residential data confidentiality the cases of microcephaly were plotted in the respective municipality’s centroids, which is a point in the middle of the municipality equidistant to its territorial limitsA microcephaly case is a confirmed case of microcephaly and or Central Nervous System alteration in new-borns (less than 28 days) and those older than 28 days. Neonatal deaths were not considered in the study.

The study area criteria selection was to be a State of the Northeast Region of Brazil (the most affected by Zika) and have a significant number of cases of microcephaly in epidemic period of 2015 to 2017. Three States selected were: Rio Grande do Norte (RN), Paraíba (PB) and Bahia (BA).

In these three States specialized care units georeferencing was made according to the protocols of the Health Departments of each State. These protocols had the specific objective of guiding actions for the care of women of childbearing age, pregnant women and puerperal women and new-borns with microcephaly related to ZIKV infection.

The data of specialized health units of each protocol were georeferenced from the address information contained in the documents. These units were established by the health secretariats of Paraíba (PB), Bahia (BA) and Rio Grande do Norte (RN).

A search on the Mymaps platform (Google) was performed to identify the respective geographical coordinates, later, these coordinates were georeferenced and transformed into a layer of the geographical information system (GIS), using the ArcMap 10.5 geoprocessing software. The variables and data sources used in this study are described in Table 1.

**Table 1:** Variable and data sources used.

Variables	Year	Utilization	Source
Confirmed cases of microcephaly, by mother’s place of residence	2015 to 2017	Distribution and analysis of confirmed cases in national territory (states/counties)	Public Health Event Records–PHER/Brazilian Ministry of Health
Health units responsible for clinical management and care, diagnosis and monitoring of pregnant women with foetuses diagnosed with microcephaly and new-borns with microcephaly	2015 (RN, PB) and 2016 (BA)	Distribution of care units to pregnant women, foetuses and new-borns affected by virus Zika	Protocols for response to microcephaly related to ZIKA infection of State Health Offices (RN, PB, BA)
Ultrasound Equipment in Use	2015	Distribution and analysis of municipalities with ultrasound equipment	National Register of Health Facilities–NRHF/Brazilian Ministry of Health
Municipal network; Roads; Municipal Headquarters	2013 and 2015	Cartographic base	Brazilian Institute of Geography and Statistics

In this paper we consider that accessibility is characterized by the provision of health services, which includes geographical accessibility (distance and displacement issues), while access refers to the initial entry into the health system (health service network) representing a dimension of health performance in the health system, as well as individual factors that may limit or expand capacity for use of services offered by it [10,11].

To give an idea of the prenatal services situation on the states selected, we calculated in the year of the beginning of the epidemic the average number of live gynaecologists-obstetricians per live birth in this year.

To model geographic accessibility, we proposed in this paper to simulate a radius of influence of 100 km (buffers) around the specialized health units in each state. This radius was chosen as a proxy of the longest possible round-trip distance for displacements of parents and children in the same day (each journey taking about 2 hours’ time by paved road). By these procedures we can estimate the municipalities that are too far from the needed health services for the children affected by ZCS.

### Ethics statement

This research is part of the project “Health promotion in the context of the Zika epidemic: actors and scenarios in decision-making processes” funded by the Zikalliance Consortium and approved by the Institutional Review Board of the National School of Public Health/Oswaldo Cruz Foundation (CEP / ENSP N. 67311617.8.0000.5240).

## Results

Studies have shown that the entry of the ZIKV in Brazil occurred in Pernambuco during the Confederations Cup in 2013 and then spread to other states [12]. The socio-sanitary conditions of the Northeast populations were pointed out as possible explanation for the high prevalence of Zika observed in this region during the epidemic period (2015–2017) [13].

According to data obtained from the Public Health Event Records–PHER/Brazilian Ministry of Health (2018) Brazil accumulated 2639 confirmed cases of microcephaly of Zika between 2015 to 2017 (327 probable cases and 1628 cases under investigation). Other malformation problems are believed to have occurred and were not reported as cases related with ZIKV infection, such as vision problems in new-borns.

After two epidemic waves of Zika, the incidence was reduced to very low values, leading to the belief that ZIKV transmission was virtually interrupted in the country, nonetheless it may still last a few years [14].

In 2015 it was already possible to note that the highest concentration of cases of microcephaly associated with ZIKV infection were in the Northeast of Brazil, with 89.8% (646) of the total confirmed cases (Table 2). The states of Pernambuco, with 236 confirmed cases and Bahia with 123 confirmed cases, were the states with the largest number of cases in the region.

**Table 2:** Number of cases reported, confirmed and discarded by Geographic Regions of Brazil from 2015 to 2017.

Geographic Regions	Notified Cases	(%)	Confirmed Cases	(%)
**2015**				
Northeast	3407	82,7%	646	89,8%
Southeast	362	8,8%	49	6,8%
North	104	2,5%	3	0,5%
Midwest	221	5,4%	19	2,6%
South	25	0,6%	2	0,3%
**Total (2015)**	4119	100%	719	100%
**2016**				
Northeast	4943	57,5%	897	61,1%
Southeast	2285	26,5%	324	22,1%
North	517	6,0%	80	5,4%
Midwest	592	6,9%	136	9,2%
South	267	3,1%	32	2,2%
**Total (2016)**	8604	100%	1469	100%
**2017**				
Northeast	935	35,3%	83	45,8%
Southeast	1035	39,1%	38	21,0%
North	236	9,0%	25	13,8%
Midwest	312	11,8%	28	15,5%
South	127	4,8%	7	3,9%
**Total (2017)**	2645	100%	181	100%
**Total of all years**	15368		2639	

**Source**: Public Health Event Records—PHER/Brazilian Ministry of Health (2018)

The peak of the epidemic was in 2016, with 1,469 confirmed cases of microcephaly in Brazil, of which 61.1% (897) occurred in the Northeast Region. In 2017 the epidemic lost strength, with 181 confirmed cases of microcephaly, of which 45.9% (83) occurred in the Northeast.

The end of the PHEIC by the ZIKV epidemic was decreed by WHO in November 2016, marking the considerable reduction in new cases. In 2017 there was a 90.7% reduction in cases in the Northeast when compared to the previous year, and the Southeast also registered a sharp 88.3% drop (Table 2).

**Fig 1:**
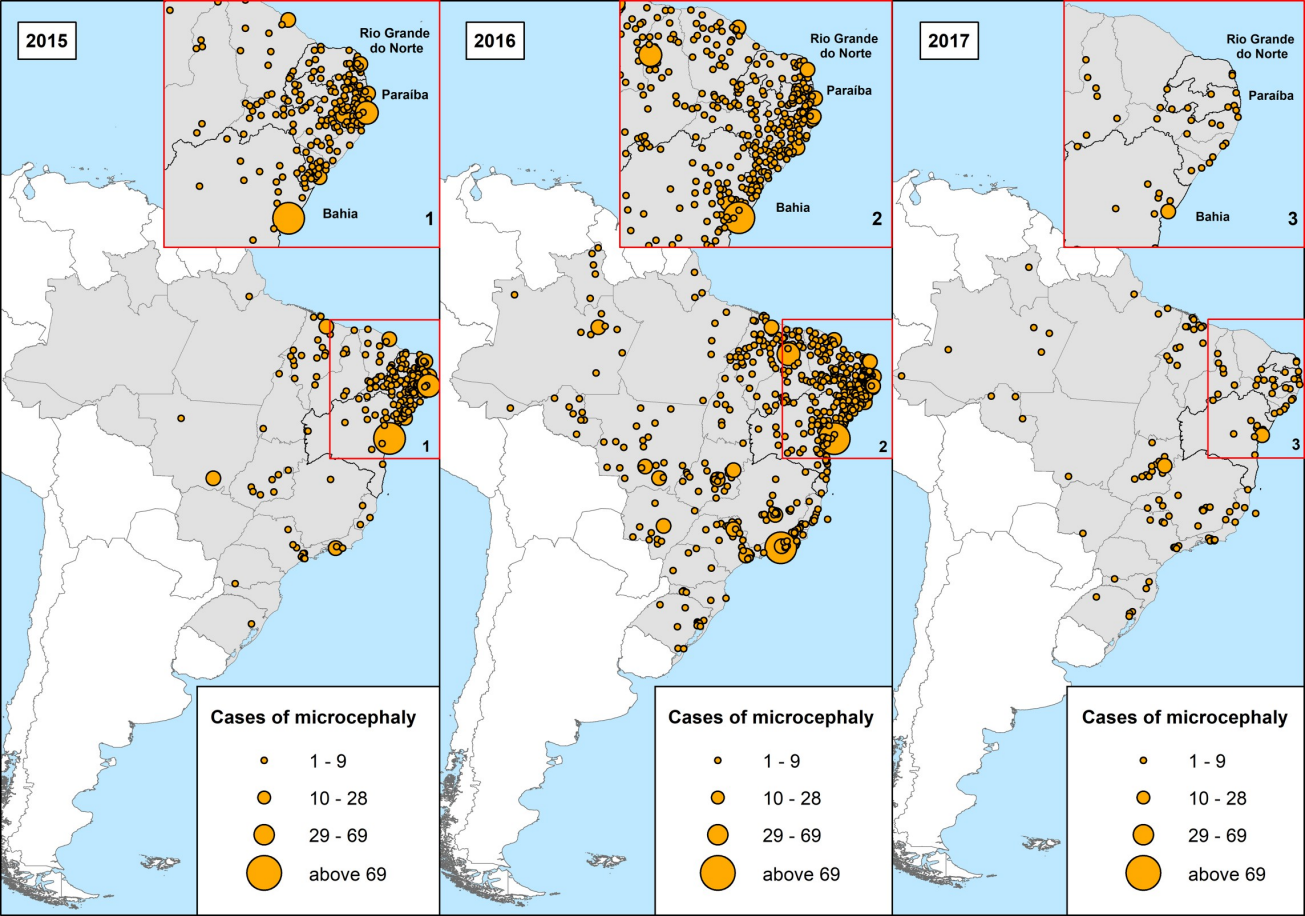
Confirmed cases of microcephaly by ZIKA, by Brazil, by mother’s place of residence, 2015–2017. Source: maps generated by LDP/IOC/FIOCRUZ based on data provided by PHER/Brazilian Ministry of Health.

Comparing spatial distribution of microcephaly cases by the mother's place of residence from 2015 to 2017 (Fig 1) reveals a significant concentration of cases on the Northeast coast in 2015 and 2016, especially in the capitals Salvador (State of Bahia) and Recife (State of Pernambuco). In 2016 ZIKV spread rapidly throughout the country, with a consequent increase in the number of cases of microcephaly. However, in 2017 the epidemic decreased.

**Fig 2:**
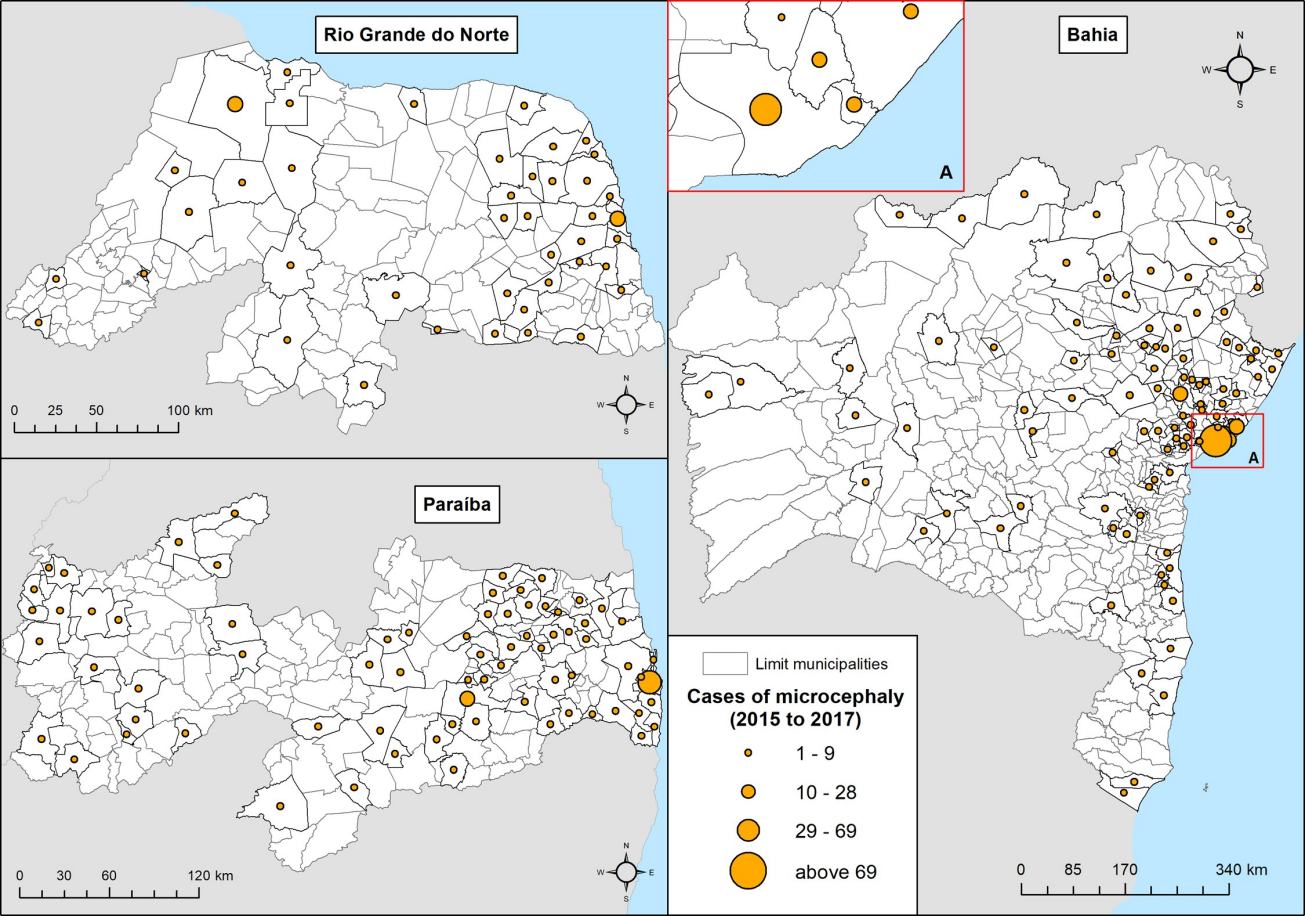
Confirmed cases of microcephaly by ZIKV, in the states of Rio Grande do Norte, Paraíba and Bahia in the period from 2015 to 2017. Source: maps generated by LDP/IOC/FIOCRUZ based on data provided by PHER/Brazilian Ministry of Health.

Among the three states selected for this study, the most critical situation occurred in the state of Bahia, with 425 confirmed cases of microcephaly in the period analysed (2015 to 2017), followed by Paraíba with 175 cases and Rio Grande do Norte with 100 cases. Like other Brazilian states, the highest concentration of microcephaly cases occurred in the respective capitals. The capital Salvador—BA had the largest number of confirmed cases (216 cases), followed by João Pessoa—PB (45 cases) and Natal—RN (26 cases). However, it should also be mentioned the significant diffusion of cases in other cities in the interior of these states. In Paraíba, for example, 33.0% of the municipalities recorded at least one case of microcephaly by ZIKV, as well as 25.1% of the municipalities in Rio Grande do Norte and 23.0% of the municipalities in Bahia (Fig 2).

In addition, by analysing the prevalence of microcephaly cases by 1,000 live births between 2015 and 2017, it is possible to have a broader idea as to the risk of having ZCS in this period. Among the three states, the worst situation occurred in Paraíba, on average, for every thousand live births 1.0 had microcephaly due to ZIKV. In the states of Bahia and Rio Grande do Norte, the situations were similar, as for every thousand live births 0.7 had microcephaly due to ZIKV.

Among these State capitals the worst situation occurred in Salvador—BA, which for every thousand live births 2.0 had microcephaly and João Pessoa—PB, where for every thousand live births 1.2 had ZCS in the study period.

From the beginning of the epidemic in 2015, the number of registered cases increased rapidly, which was partially attributed to better notification, especially among pregnant women, and the possibility of sexual transmission of the virus. Nevertheless, underreporting trends remain, due to the high percentage of asymptomatic cases, low demand for care, and difficulties in differential diagnosis compared to other febrile diseases [15,16].

As far as attention to pregnancy and childbirth care, a major concern for Zika surveillance and ZCS diagnosis, the primary health care network in the three states selected have many difficulties to meet its requirements as it does not have the necessary means in personnel, technical support and territorial coverage.

Bahia has the largest number of gynaecologists-obstetricians—947 for a total of 206,655 live births in 2015(218 live births per gynaecologists-obstetrician on average). The municipality of Senhor do Bonfim (located in the State’s northeast region), with 1,107 live births per obstetrician is the one in the worst situation. In contrast, in the municipality of Dom Macedo Costa the rate was of only 46 live births per obstetrician (this municipality is 2h30 from Salvador).

The State of Paraíba, with 306 gynaecologists-obstetricians had 59,089 live births in 2015, an average of 164 live births per gynaecologist. The highest average number of live births per obstetrician occurred in the municipality of Santa Rita with 2,141 live births per physician (the municipality with about 130,000 inhabitants disposed of only one obstetrician- gynaecologist). The best situation occurred in the municipality Cabedelo with an average of 59 live births per physician.

Finally, in Rio Grande do Norte there were 251 gynaecologists-obstetricians and an average of 196 live births per physician. The highest rate occurred in the municipality of Extremoz with 568 live births per physician, and the lowest value in the state capital of Natal with 77 live births per physician.

The follow-up of pregnancy requires the use of diagnostic support equipment, such as ultrasound for the correct diagnosis of foetal malformation, such as those caused by Zika. However, we found out that many Brazilian municipalities don´t have this equipment and it is thus often necessary to travel long distances to have access to these tests. In in the year of 2015 about 35% of the municipalities didn’t had ultrasound equipment, but this percentage varied from state to state.

Among the three states of this study there is a greater availability of ultrasound equipment in Bahia, with 76% of the municipalities properly equipped, while in Paraíba and Rio Grande do Norte this percentage falls to 65% and 63%, respectively.

Prenatal consultation coverage is also quite uneven in the study area. If we take the year of 2016 as reference, the best prenatal consultation coverage occurred in the State of Paraíba, followed by Rio Grande do Norte and Bahia.

The access to specialized referral units and professionals for the treatment and rehabilitation of ZCS cases are distributed according to health regions. Health actions, services, resources and technology are organised in Health Care Networks (HCN). (Ministry of Health, Ordinance No. 4.279, of 12/30/2010).

The HCN establish service flows, actions and resources focused on the care and development of the children and their families, thus playing a role of articulating and accompanying, together with other actors, in order to reduce morbidity and mortality in places of difficult access or scarcity of health resources. The role of HCN is important for comprehensive care (treatment and rehabilitation), especially in situations where large distances may reduce access to services [17].

In the State of Bahia, according to the protocols of care and monitoring of children with ZCS, there were 13 units qualified to provide specialized services for these children, but they did not cover all the territory which has 28 HCN [7].

Nonetheless, among the three States of this study, Bahia (a State about the size of Spain) is the one with the largest number of specialized services for children with ZCS. On the other hand, they are largely concentrated in the eastern region of the state, making access to services from other regions difficult due to long travel distances.

**Fig 3:**
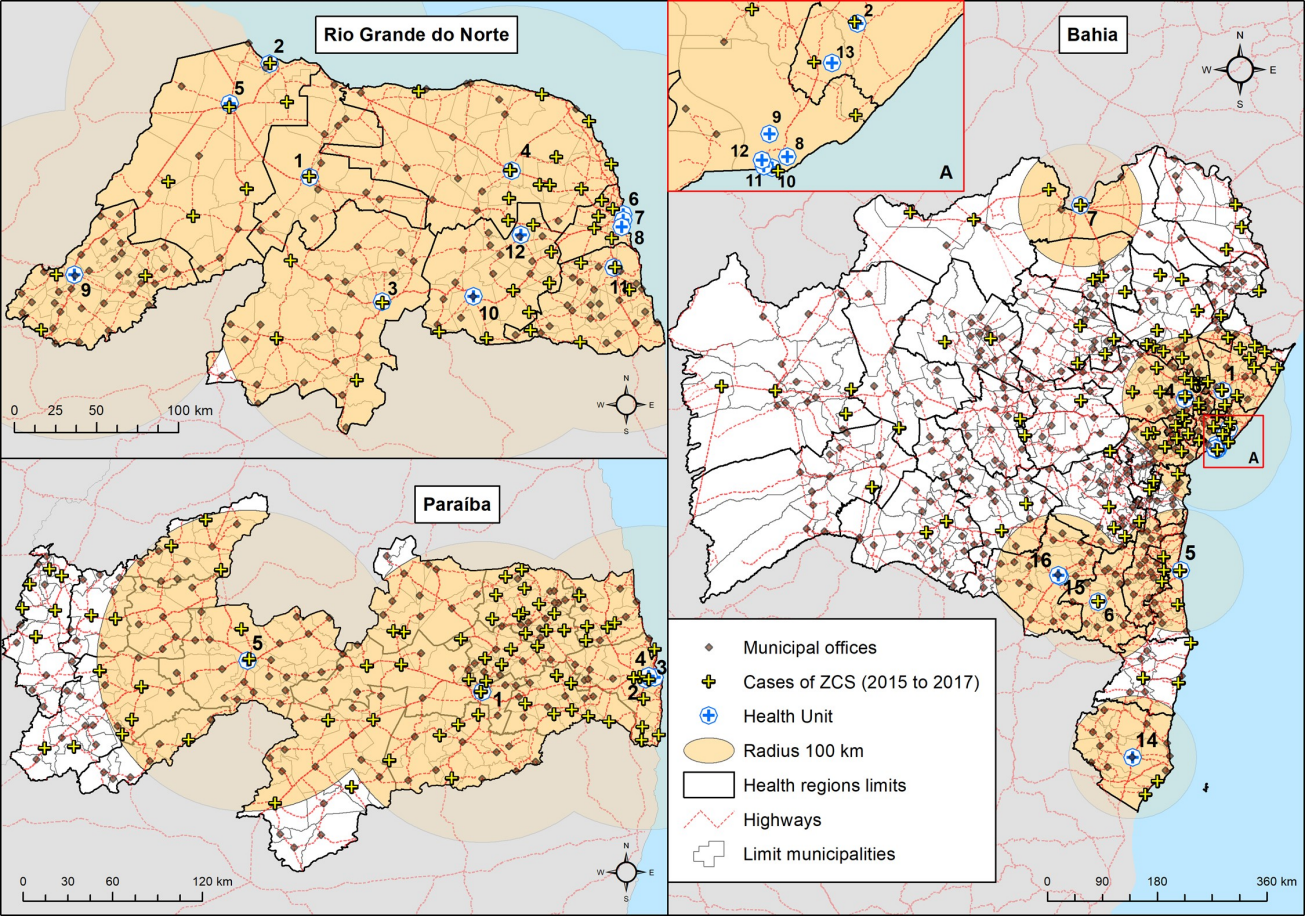
Municipal headquarters located within 100 km of the microcephaly care units in the states of Rio Grande do Norte, Paraiba and Bahia. The names of the respective health units are shown in Table 3. Source: maps generated by LDP/IOC/FIOCRUZ based on data provided by State Health Department of Rio Grande do Norte; Paraiba State Department of Health; State Secretariat of Health of Bahia.

The State of Paraíba (ten times smaller than Bahia) has only 5 specialized healthcare units for a total of 16 HCN [18]. The State of Rio Grande do Norte (almost the same size as Paraíba) was fully covered by one specialized care unit in every HCN [18,19] (Fig 3).

**Table 3:** Health units indicated in the respective care and follow-up protocols for children with ZCS.

States	Municipalities	Care units for children with microcephaly	Services
**Rio Grande do Norte**	Açu	**1 –**Integrated Rehabilitation Center	Monitoring and development of children with microcephaly by a multiprofessional team
	AreiaBranca	**2 –**Rehabilitation Center of Areia Branca	
	CurraisNovos	**3 –**Rehabilitation Center Prof. Crindelia Bezerra	
	João Câmara	**4 –**Childhood and Adult Rehabilitation Center	
	Mossoró	**5 –**APAE	
	Natal	**6 –**Universitary Hospital Onofre Lopes	
	Natal	**7 –**Child Rehabilitation Center	
	Natal	**8 –**Potiguar Center for Neurofunctional Rehabilitation	
	Pau dos Ferros	**9 –**Specialized Rehabilitation Center	
	Santa Cruz	**10 –**Specialized Rehabilitation Center	
	São José de Mipibu	**11 –**Specialized Rehabilitation Center	
	São Paulo do Potengi	**12 –**Geraldo Felix Santa Rosa Child and Adult Rehabilitation Center	
**Paraíba**	Campina Grande	**1 –**Elipidio de Almeida Health Institute	Diagnostic investigation and monitoring of growth and development of new-borns diagnosed with microcephaly
	João Pessoa	**2 –**Father Damião Maternity	
	João Pessoa	**3 –**Lauro Wanderley University Hospital	
	João Pessoa	**4 –**Candida Vargas Maternity	
	Patos	**5 –**Dr. Peregrino Filho Maternity	
**Bahia**	Alagoinhas	**1 –**Pestalozzi Society	Monitoring and development of adults and children with physical and intellectual disabilities
	Camaçari	**2 –**Multiprofessional Center for Physical Rehabilitation	
	Feira de Santana	**3 –**APAE	
	Feira de Santana	**4 –**APAE	
	Ilhéus	**5 –**Specialized Service Center	
	Itapetinga	**6 –**APAE	
	Juazeiro	**7 –**Prevention Center Rehabilitation and Social Inclusion	
	Salvador	**8 –**CEPRED	
	Salvador	**9 –**Sister Dulce Social Works Association	
	Salvador	**10 –**Child Care Center with Cerebral Palsy	
	Salvador	**11 –**Bahia Institute of Neurological Organization	
	Salvador	**12 –**Physical and Mental Rehabilitation Clinic	
	Simões Filho	**13 –**Sister Dulce Social Works Association	
	Teixeira de Freitas	**14 –**Mother Mary Physical Rehabilitation Center	
	Vitória da Conquista	**15 –**Municipal Center Specialized in Physical and Hearing Rehabilitation	
	Vitória da Conquista	**16—**APAE	

**Fig 4:**
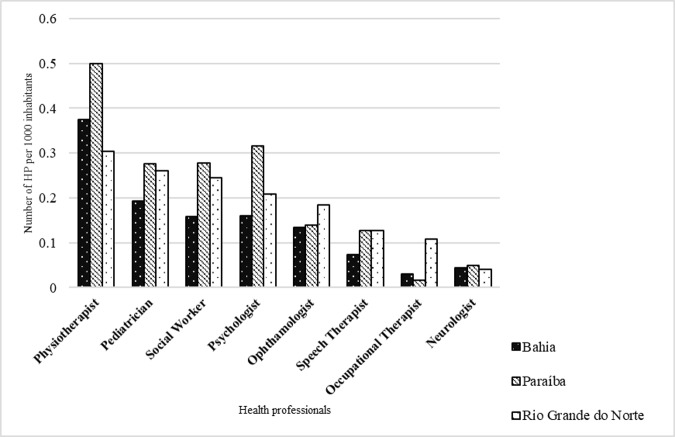
Number of health professionals for every thousand inhabitants in Bahia, Paraíba and Rio Grande do Norte, 2015–2017. Source: National Register of Health Facilities–CNES/Brazilian Ministry of Health (2020).

In these specialized units, health professionals responsible for the rehabilitation and early stimulation processes of children diagnosed with microcephaly by ZIKV are expected to be found. However, the analysis of the number of professionals per thousand inhabitants (considering the population of the respective states and taking into account that these professionals are not restricted to caring only these children), pointed out that none of the states of the study area had at least one specialized doctor for every thousand inhabitants (Fig 4).

In the State of Bahia the most available health professionals were the physiotherapists (0.37 per thousand inhabitants), followed by pediatricians (0.19), psychologists (0.16) and social workers (0.16). On the other hand, the professionals least available were occupational therapists (0.03), neurologists (0.04) and speech therapists (0.07). In Paraíba, physiotherapists were also the most available professionals (0.5) followed by psychologists (0.32), social workers (0.28) and pediatricians (0.28).

As in Bahia neurologists (0.05) and occupational therapists (0.02) were least available. in the situation in Rio Grande do Norte was also similar, with a better availability of physiotherapists (0.30), followed by pediatricians (0.26), social worker (0.24) and psychologist (0.21The least available professional were neurologists (0.04).

## Discussion

The context of the Zika epidemic since the detection of the first cases of microcephaly in Pernambuco, demonstrated the importance of health surveillance and the availability of health professionals who worked to identify the first cases in November 2015, which corroborate with our findings [20].

It is not yet clear why the Northeast Region had such a high number of microcephaly cases, as ZIKV spread with great speed throughout the country in 2016 [13]. Outside the Northeast Region only isolated cases were detected in the beginning of the epidemic, which may be due to underreporting before the declaration of Public Health Emergency of International Concern, by the Ministry of Health of Brazil and WHO in 2016 and the fact that the compulsory notification of Zika was only initiated in February 2016 (Ministry of Health / Ordinance 204 of 02/17/2016).

A study by the Ministry of Health evaluated the responses of the Brazilian Public Health System to the Zika epidemic, pointing out the importance of women's access to healthcare services for the early diagnosis of problems in pregnancy and childbirth, as well as to the treatment and rehabilitation of children born with ZCS [3]. However, it was observed many fragilities on pregnancy follow-up in the Primary Care System in most Brazilian municipalities, which corroborates our findings about the low coverage of routine prenatal follow up (below WHO recommendations) in most municipalities of the study area, as well as the lack of diagnostic imaging (ultrasound equipment), indispensable for monitoring and diagnosing problems in pregnancy. The identification of health and social needs of children and their families, aiming at health promotion, are among the activities carried out by the Family Health Strategy (FHS) teams for maternal and child health that can have a positive impact in addressing the Zika epidemic. It is the role of the FHS teams to advise pregnant women and encourage prenatal consultations, with early detection of congenital changes through imaging (ultrasound) and early identification of patients most likely to have an unfavourable evolution [7].

In our study we found a low coverage of ultrasound equipment in the municipalities of the States of Bahia, Paraíba and Rio Grande do Norte, which points to an increased vulnerability of these populations to the consequences of Zika infection during pregnancy.

On the other hand, we have found that microcephaly cases were highly concentrated in the State capitals which are the municipalities best provided by health units and equipment such as ultrasound as well as they have a better coverage of health professionals.

However, Mendes et al. (2019) [21] show that there is a deficit of information regarding reproductive health and rights that the health system could not solve and that would have been fundamental for women at risk of Zika. The latter point out that many mothers only found out that their children had ZCS at delivery or later and discuss the difficulty of health professionals in properly communicating a diagnosis so impactful on the life of the child and their families.

Equally difficult, is to adapt the family to a new routine of consultations and referrals until a treatment protocol can be established that involves a wide range of professionals and services, most often located very far from the affected families residences, or even worse, in other cities.

In our study we found that there are large areas uncovered by accessible specialised health care services and consequently by health professionals like neurologists and occupational therapists, especially in Bahia and in Paraíba.

Furthermore, the Brazilian ZIKV epidemic has generated experiences of social inequities in access to rights and revealed unfortunate scenarios, such as pregnant women infected by the virus thus causing the birth of children with unexpected health needs, marked bodies, and tests that uncovered a non-standard neurological system, even for known microcephalies, resulting from other congenital diseases. This highlights stories, especially, of poor Northeastern women, who have organized themselves to demand responsibility from the Brazilian Government for the epidemic and its consequences to the lives of their children [22].

These mothers understand that their children born with microcephaly are also responsibility of the State, because of its failures in preventing these cases, and thus it now must offer rehabilitation, schooling for affected children and the prompt offer of social benefits.

From the point of view of health care, Moreira and collaborators (2018) [22] point out that children with Zika Congenital Syndrome (ZCS), who bear in their bodies the marks of multiple disabilities and chronic and complex health conditions, require a multidisciplinary team for their care, including physiotherapists, nurses and nursing technicians, speech therapists, nutritionists, educators, social workers, occupational therapists and psychologists. It is an irrefutable need for these children who require proper organization of these teams for their care. It should be noted that pediatricians are not the only doctors involved in treating children living with chronic and complex health conditions. For example, children with ZCS need ophthalmologists due to eyesight problems, as well as neurologists due to the convulsions and neurological morbidities that commonly accompany the disease.

There are some limitations to this study as the missing data of Zika cases. Despite advances and rapid response from the scientific community and the Brazilian National Health System–the Unified Health System (UHS) to the epidemic, there are still gaps in coverage and completeness of Zika and ZCS case records. The very first case definitions of microcephaly, which at the beginning of the epidemic were based solely on head circumference measurement, had to be reviewed. Research findings showed that other signs and symptoms could be indicative of central nervous system malformations caused by the ZIKV.

Data about Zika was also affected by the occurrence of a triple epidemic of dengue, Zika and chikungunya. These are diseases whose symptoms can be confounded, making diagnosis more difficult, especially due to the novelty of the ZIKV in Brazil at that time, and the fact that specific diagnostic kits were not yet available.

Another limitation to this study is that it considered for the accessibility analysis only public health establishments. The methodology to estimate geographical distance between health services and patients, the radius of 100 k, doesn´t correspond to the real distance, as it didn´t considered the actual routes and roads used by these patients, this radius was used as a proxy for the study.

## Conclusions

The Zika and microcephaly epidemics in Brazil pose important challenges for health services and require the optimization of the relationship between primary care and specialized services. The nation’s large expanse, and its high vulnerability to the epidemics, which are largely due to institutional weakness, has resulted in leaving large portions of the country with low service coverage (specialized equipment and human resources). This constitutes a highly heterogeneous picture regarding access to the services required for diagnosis, follow-up, treatment, and rehabilitation of affected mothers and children.

It is worth mentioning that geographical accessibility by itself does not guarantee access to health care because it also depends on local and individual characteristics. Thus, the findings of this study need to be complemented by qualitative research that addresses the other dimensions of access as pointed out in other studies [10,11].

The consequences of the Zika epidemic are particularly heavy for affected children and their families and needed to be addressed by the National Public Health System through establishing viable flows between the demand and supply of specialized services for these children and families.

Intercity and interstate flows, even temporary, act as an individual alternative for access to more specialized health services. In the area studied, cities with greater resources, mainly state capitals, stand out. Therefore, they become places of confluence of families in search of specialized attention and rehabilitation for children born with microcephaly and other health problems.

The distances between the mothers' and children's homes to the health care providers listed in the States health care protocols of the study area, as well as their territorial dispersion, lead to multiple displacements of these patients and their caregivers. This leads to extreme physical and mental distress, as well as impeding work, and the ability to perform other activities of daily life.

This situation is even worse when it comes to families living in peripheral areas who depend on public services for care (including transportation to health facilities). The current prospects for cuts in public health posed by the approval of PEC 55/2016 (Constitutional Amendment) and the growing precariousness of labour relations in Brazil indicate there are going to be increasingly difficult situations for these families and children affected by the epidemic, covered only by the consolidation of social support networks of mothers and family members, even though there are guarantees provided by legislation such as the Constitutional right to health for the entire population.

The first children affected by ZCS are currently three years of age and older, and new needs are emerging that should provide for by the UHS (National Health System). Due to this situation, after numerous threats of Continuous Instalment Benefit (CIB) cuts (governmental aid to vulnerable populations), a project was presented to the Congress that provides for the creation of a lifetime pension for children born with ZCS, thanks to the large mobilization and support of organizations of parents with children with ZCS and other social movements.

In this sense, access to health is more than simply a matter of the relationship between supply and demand for health care, it is built on the active participation of the population as a way of stimulating dialogue between the health situation and the territory. The Health Promotion approach configures a new cultural perspective, broadening the identification and discussion of social determinants of health (SDH), giving rise to adequate responses to the reality experienced by the subjects in solving collective problems [23,24]. Thus, it represents and promotes a possible cultural transition, the establishment of a pro health culture and a sense of belonging of the population to a set of practices and behaviours presented by the institutional agents, as well as the respective alternatives of rearrangements in the societal logic structured by Health Promotion.

Finally, it is the State’s responsibility to expand public policies aimed at controlling arboviruses like Zika, which until the present have focused merely on combating vector and focal environmental control measures. These measures have been insufficient in eliminating vector borne diseases transmitted by the *Aedes aegypti* mosquito and there should be further initiatives, as part of a health promotion program through inter-sector actions (Conditional cash Transfers-BFP, housing programs, educational programs for children with special needs, among others), with broad participation of organized civil society, acting on structural issues (sanitation, water supply, public transportation, etc.) not only reducing risk and exposure but also reducing the general population’s vulnerability.

## Supporting information

S1 Graph (XLSX)
